# FISHing for Answers in Postoperative Atrial Fibrillation

**DOI:** 10.1161/JAHA.112.002931

**Published:** 2012-06-22

**Authors:** Barry London

**Affiliations:** Division of Cardiology, University of PittsburghPittsburgh, PA

**Keywords:** editorials, atrial fibrillation, arrhythmias, cardiac, fatty acids, omega-3, heart surgery

Dietary fish intake and ingestion of omega-3 polyunsaturated fatty acids (n3-PUFAs) have been associated with lower rates of ventricular and atrial arrhythmias in some (but not all) observational studies and randomized controlled clinical trials performed on diverse human populations.^1,2^ These findings have sparked detailed investigations to identify the potential antiarrhythmic mechanisms. In vitro studies have shown that n3-PUFAs affect cellular membrane properties, ion channels and exchangers, nuclear receptors, transcription factors, kinases, and inflammatory mediators. In vivo studies have identified potential antiarrhythmic effects from short- and long-term treatment with n3-PUFAs in small and large animal models.^3–5^

Several single-center studies have examined whether n3-PUFA treatment decreases the incidence of atrial fibrillation (AF) after cardiac surgery (Table 1).^6^ In an open-label study in 2005, Calò et al^7^ reported a 64% decrease in postoperative AF in patients treated with 1.7 to 1.8 g/d of docosahexaenoic acid / eicosapentaenoic acid given 5 days preoperatively through discharge versus those treated with usual care (n=160, *P*=0.01). Three other double-blind, placebo-controlled studies in which 2.0 to 4.6 g/d of docosahexaenoic acid / eicosapentaenoic acid was started 5 to 21 days before the operation and continued until discharge reported no difference (n=170, *P*=0.99), a 66% increase (n=108, *P*=0.28), and a 37% decrease (n=200, *P*=0.11) in postoperative AF, respectively.^8–10^

**Table 1.** Trials of N3-PUFAs in Postoperative Atrial Fibrillation

Study	Surgery	Dose	Duration	AF End Point	N	AF Controls, %	Odds Ratio
Calò et al (2005)^7^	CABG	1.7–1.8 g/d EPA/DHA 1:2 vs Control	After surgery to discharge	≥5 min or Rx	160	33	0.36 (*P*=0.01)

Heidarsdottir et al (2010)^8^	CABG and/or valve	2.2 g/d EPA/DHA 1.2:1 vs 2.0 g/d olive oil	5–7 d before surgery to discharge	≥5 min	170	54	1.0 (*P*=0.99)

Saravanan et al (2010)^9^	CABG	2.0 g/d EPA/DHA 1.2:1 vs 2.0 g/d olive oil	7–15 d before surgery to discharge	≥30 s	108	43	1.7 (*P*=0.28)

Farquharson et al (2011)^10^	CABG and/or valve	4.5 g/d EPA/DHA 1.4:1 vs sunflower oil	21 d before surgery to 6 d after surgery or discharge	≥10 min or Rx	200	48	0.63 (*P*=0.11)

FISH Trial, Sandesara et al (2012)^11^	CABG and/or valve	4.0 g/d before surgery and 2.0 g/d after surgery EPA/DHA 1.2:1 vs corn oil	≥1.5 d before surgery to 14 d after surgery	AF requiring drug Rx, cardioversion, or a-pacing	260	33	0.89 (*P*=0.67)

OPERA Trial, Mozaffarian et al (2011)^12^	All cardiac surgery except ventricular assist device / transplantation	10.0 g in 3–5 d or 8.0 g in 2 d before surgery and 2.0 g/d after surgery EPA/DHA vs olive oil	2–5 d before surgery to 10 d after surgery or discharge	≥30 s	1516 target	N/A	N/A

AF Controls indicates percentage of control subjects who meet end point criteria for AF or flutter; a-pacing, atrial overdrive pacing; EPA, eicosapentaenoic acid; DHA, docosahexaenoic acid; N/A, not available; Rx, treatment; and Valve, cardiac valve repair or replacement.

In this issue of the *Journal of the American Heart Association (JAHA)*, Sandesara et al^11^ report the results of a multicenter, randomized, placebo-controlled trial of 260 patients given 4.0 g/d n3-PUFA versus corn oil from 1.5 days before until 2 weeks after coronary artery bypass grafting with or without concomitant valve surgery. The primary end point, unlike prior studies, was clinically significant AF, defined as AF requiring pharmacological therapy, pacing, or cardioversion. Plasma n3-PUFA levels increased significantly in the treatment group. The incidence of AF was decreased by a nonsignificant 11% in the n3-PUFA group (*P*=0.67), and the authors concluded that the study does not support a beneficial effect of n3-PUFAs in the prevention of postoperative AF.

Postoperative AF is an appealing target for studies of AF treatments for several reasons. Patients are monitored closely for rhythm disturbances during the early postoperative period, and the incidence of AF in the 2 weeks after cardiac surgery exceeds 30%. Most of the patients who have cardiac surgery have traditional risk factors for AF. In addition, myocardial/pericardial inflammation is an important contributor to postoperative AF, and n3-PUFAs are thought to specifically affect these pathways.^1,2^ Of course, these findings may have limited relevance for AF in other settings.

Sandesara et al suggest several explanations for the disparate results of the randomized studies that have looked at postoperative AF. These include the relatively small sample sizes of the trials, differences in drug duration and dosage, differences in types of cardiac surgery, variation in the length and intensity of follow-up, and differences in the definition of postoperative AF. All of these are valid concerns. In addition, another potential set of confounding variables should be considered. Preoperative and postoperative medical therapies have evolved during the past decade. Most cardiac surgical patients now take antiplatelet/antiinflammatory therapies (aspirin, clopidogrel), β-adrenergic–blockers, angiotensin-converting enzyme blockers or angiotensin receptor blockers, and cholesterol-lowering agents such as statins that have pleiotropic antiinflammatory actions. It is possible that the positive effects seen in earlier arrhythmia trials with n3-PUFAs are damped by the use of these pharmacological agents with proven efficacy in decreasing the morbidity and mortality rates of coronary artery disease and its complications.

The Omega-3 Fatty Acids for Prevention of Post-Operative Atrial Fibrillation (OPERA) trial is a multinational, randomized, double-blind, placebo-controlled trial of n3-PUFAs versus olive oil, with a target enrollment of 1516 patients and 90% power to detect a 25% decrease in postoperative AF, given an event rate of 30% in controls.^12^ One might expect this trial, which will enroll more patients than all of the prior trials combined, to definitively answer the question of the efficacy of n3-PUFAs for postoperative AF (Table 1). Given the history of trials that have used n3-PUFAs for atrial and ventricular arrhythmias, however, this might be overly optimistic.

In summary, Sandesara have added another important study to the literature that challenges the antiarrhythmic benefit of n3-PUFAs. Their findings are truly relevant only for postoperative AF, and a more definitive answer to this specific clinical scenario may come from the OPERA trial. If the results hold true, though, we should consider whether the underlying assumption of efficacy of n3-PUFAs for arrhythmia prevention is “fishy.” Are n3-PUFAs efficacious in the setting of the myriad other pharmacological and interventional therapies in the modern cardiologist's arsenal? Time and additional studies will tell.
